# Introducing electric spring in the voltage frequency regulation of a multi area multi source integrated power system network

**DOI:** 10.1038/s41598-025-05576-y

**Published:** 2025-07-01

**Authors:** Debdeep Saha, Soham Dutta

**Affiliations:** 1 Grid Edge Solutions, IDC, Hitachi Energy Services Pvt. Limited, Bengaluru, India; 2 Department of Electrical and Electronics Engineering, Manipal Institute of Technology, Manipal Academy of Higher Education, Karnataka, 576104 India

**Keywords:** Automatic generation control, Cross - Coupling, Electric spring, Golden Jackal optimization, Random load perturbation, Electrical and electronic engineering, Renewable energy

## Abstract

The present work demonstrates the application of an electric spring in a multi-source interconnected power system in which coordinated control of frequency and voltage loop is investigated. A two-area power system is considered with non–linearity for the thermal power system, such as generation rate constraint and governor dead band, so that the developed model is realistic. A novel cascade controller, namely a two–degree–of–freedom proportional–integral–derivative controller cascaded with a proportional–integral–derivative controller (2DOFPID–PID), is utilized for the first time for reducing the area control error to zero in both the control areas. A powerful algorithm known as the Golden Jackal Algorithm (GJA) is considered for tuning the controller parameters and achieving the minimum performance index. System dynamic responses are observed for the coordinated automatic load frequency control and automatic voltage regulator during step load perturbations and random load perturbations. Selection of the best performance index (PI) among integral of squared error (ISE), integral of time multiplied by absolute error (ITAE), integral of time multiplied by squared error (ITSE), and Integral of absolute error (IAE) proves that ITAE serves the best among others. To model an AC/DC bus, system responses are also investigated with a parallel AC/DC link that depicts satisfactory results in terms of overshoot, undershoot, and settling time. Modelling of the electric spring in the proposed system is integrated to check the terminal voltage deviation and frequency deviation, and mitigate them. Results show reduced generator terminal deviation in both the control areas. Comparison of different powerful algorithms with the proposed one infers the superiority of the proposed golden jackal algorithm with reference to the performance index vs. number of iterations.

## Introduction

 Amid the target of 2030 to achieve 500 GW renewable energy sources (RES) penetration in the Indian power grid, aligning with Sustainable Development Goal No. 12 (affordable and clean energy), there has been a constant reduction of inertia. Hence, there is a need to control frequency and voltage deviation under normal limits^1^. Automatic generation control provides secondary control to ensure minimum frequency and voltage deviations^2– 6^. However, the continuous growth of RES integration has put more challenges to controlling the grid inertia, which in turn affects the voltage and frequency. Converter-based approaches are common in supporting the grid frequency and voltage by tuning their inherent parameters in synchronism^7^. Moreover, similar results can be achieved by a concept of demand-side management. Hence, a new concept of electric spring (ES) replicating a mechanical spring can be employed as an interconnected AC/direct current system that takes care of the demand side management and regulates the grid terminal voltage^8^. An ES can also be treated as a voltage compensator when series connected with non–critical loads such as water heaters and air conditioners, and parallel connected with critical loads such as grid voltage.

## Literature review

Much literature has reported load frequency control on multi-area multi-source models considering diverse generating sources such as thermal, hydro, solar thermal, gas, and distributed energy sources^1–4^. Moreover, much literature also includes combined regulation of voltage and frequency in interconnected systems, including traditional and non-traditional sources. Authors in Ref. 6 have carried out the coordinated regulation of voltage and frequency in a three-area CCGT and thermal system, incorporating the nonlinear constraints such as the Generation rate constraint and the governor dead band. Another work has been demonstrated with unified voltage and frequency control considering solar thermal, thermal, and diesel generation in a single area and two areas, respectively^4– 6^. Several other studies have been reported with power quality solutions devices, Power system stabilizers, etc., with unified control of voltage and frequency^7^. These devices are utilized to maintain the frequency deviation and oscillations in a control area with reference to the generator swinging.

Another electrical device called the electric spring (ES) has been introduced, which takes care of terminal voltage fluctuation reduction. ES is based on Hooke’s law, which is the concept of a mechanical spring. ES is a power device in series^8^ which is connected in between integrated grid and dissipative load such as heating, cooling, and lighting loads, who are tolerant to fluctuating supply and referred to as non-critical load. Hence, this combination constitutes a smart load^9^. The remaining loads such as hospitals, industries, data centres are considered as critical loads. Authors have reported control strategies with electric spring to deal with the instability issues in the interconnected grid as well as in isolated grid which in turn lowers the power quality of a system^10^. Application of electric spring in a micro grid environment is also implemented considering critical and non-critical load^11^. Voltage profiles of a network is improved by usage of an electric spring using two step voltage management strategy^12^. A state space model of electric spring embedded with distributed generation is also represented in. Since its advent from 2011, many forms and structures have been proposed and industrial forms has been evaluated^13– 15^.

When ES are integrated with non-critical loads, it forms smart load. The advantages are such as (i) following the dynamics of load generation when load demand changes (ii) voltage regulation during terminal voltage change in generator^15^. It is in those situations, when large number of renewable energy sources, electric spring can be projected as device to mitigate voltage fluctuations^16– 17^. However, considering various literatures^18– 19^ it is observed that electric spring is not utilized in frequency regulation and voltage regulation in interconnected and isolated systems. One of the constituents of ES also consists of a power electronic converter for improving the power quality of the supply voltage by reducing voltage harmonics.

In a microgrid or smart grid environment where both AC and DC buses are available, there is no literature available which provides a demand side management to provide voltage support to the infinite grid^1– 6^. No work has been carried out with conventional and non-conventional sources such as thermal, gas, dish Stirling and wind turbine generator. It is a necessity to consider the system which provides a unified voltage and frequency support with the implementation of electric spring. The modelling of voltage control along with the electric spring is an interesting point of coupling to the interconnected AC/DC system^17– 19^.

In a coordinated voltage and frequency control, the implementation of controllers serves a unique requirement for regulation. In the discussed two loops considered – ALFC and AVR, there minimum number of controllers required is two (one for ALFC and one for AVR)^5– 6^. Many secondary and voltage controllers such as classical proportional–integral–derivative^20– 21^fractional order PID^22^ two – degree – of – freedom PID^23^ and cascade controller have been proposed in different literatures^24– 27^. Classical PID controller^20^ and 2DOFPID^23^ controller have proved to be successful in implementing stabilization in terms of voltage and frequency. Among all the types of controllers, cascade controllers have two loops – inner and outer loop. Outer loop acts after inner loop have reduced the error. i.e., the change in deviations. There is an edge of two controlling actions because of the two loops^26– 27^. The PID controller offers a faster response to disturbances and ensures error reduction before moving to the outer loop, which is why it is chosen as the inner loop control. However, the 2DOF PID controller offers more tuneable parameters, enhanced Setpoint Tracking, and a better rejection rate of disturbances. So, even if the error exists after the inner loop control using PUD, the outer loop employing 2DOFPID can reduce the remaining error to zero. Over and above that, in a multisource model, ALFC and AVR loop in conjunction with AC/DC link and electric spring require robust and more tunable parameters in the proposed controller for which the proposed controller 2DOFPID-PID best fits the scenario. However, no literature has reported any work considering 2DOF classical controller as an outer loop controller and classical PID as an inner loop controller in a system that implements an electric spring. To tune the controller gains, a powerful algorithm is required to achieve the minimum objective function, thereby reducing the area control error to zero^26– 27^. There is evidence of many metaheuristic algorithms being successfully utilized in multi-area multi-source systems, such as lightning search algorithm^4^ differential evolution- artificial electric field (DE-AEFA) algorithm^20^ Hybrid Salp Swarm Differential Evolution^23^ and Bat algorithm^27^. Golden Jackal Optimization, a natured inspired algorithm based on collaborating hunting behaviour of golden jackals in nature^28^. GJO has an advantage of extremely good exploration ability which helps to locate the search space initially and attain the global minima in lesser iterations^28– 30^.

In this paper, the execution of electric spring is evaluated for the first time in a coordinated control of voltage and frequency of a multi-area and multi-source AC/DC interconnected system. Small signal analysis form of Electric Spring is considered and integrated with the AC/DC system. The load connected to the grid is considered as the critical load. The role of electric spring is to mitigate the terminal voltage fluctuation across the generators connected which in turn will have an impact on the frequency deviation due to the cross-coupling effect. across the critical loads for demand side management. Hence, a generation-voltage management strategy for controlling voltage and frequency for multi – source multi area is demonstrated in the present work. A transfer function modelling of automatic voltage regulator (AVR) loop with implementation of electric spring (ES) is depicted for a two-area system consisting of a thermal, gas, dish Stirling solar thermal (DSTS), wind turbine generator (WTG). Novel cascade controller 2DOFPID – PID in secondary and voltage control is also considered whose gains are tuned by Golden Jackal Optimization technique.

## Power system investigated

**Fig. 1:**
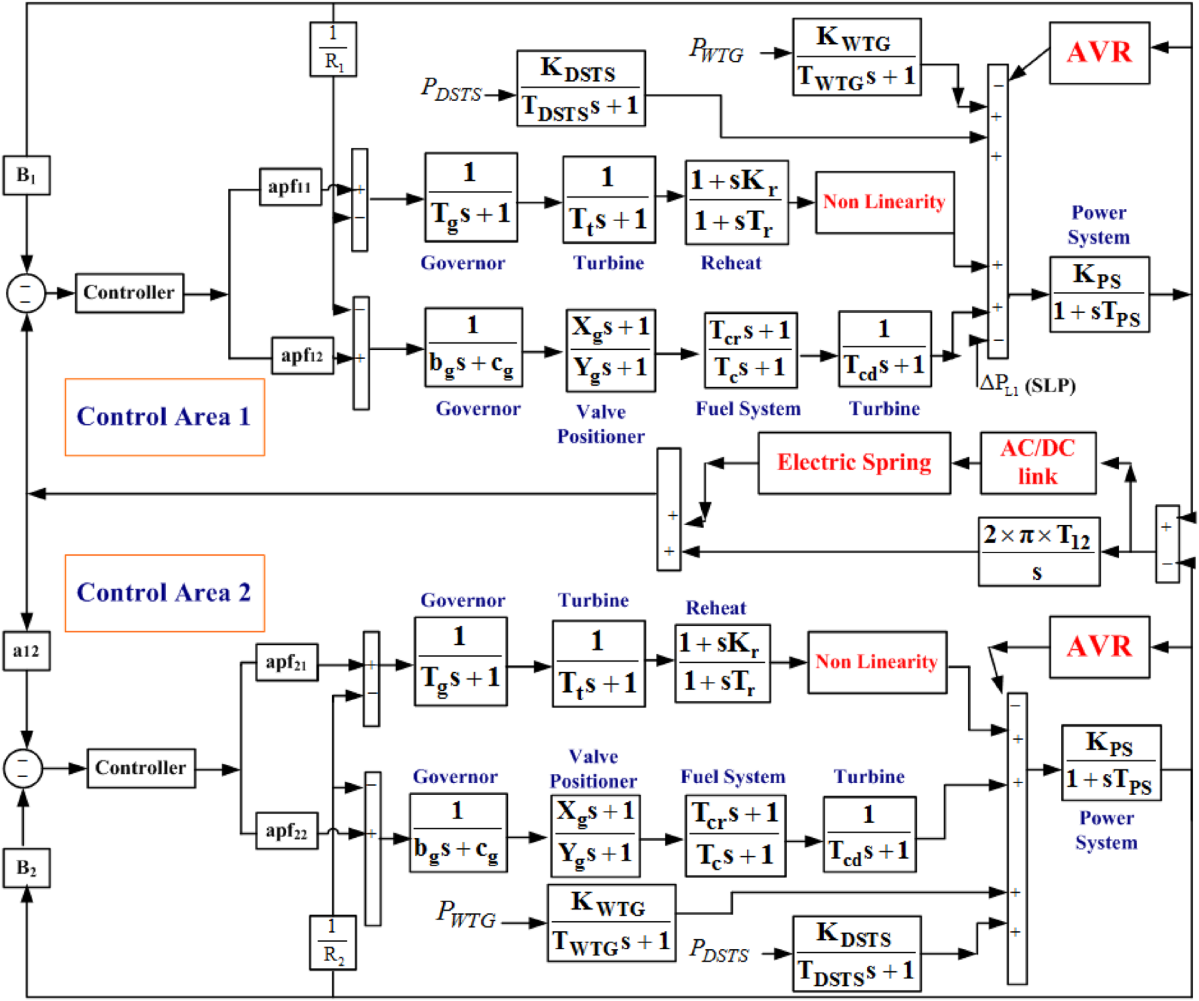
Transfer function modeling of the two-area interconnected system with inclusion of AC/direct current parallel link and Electric Spring.

A multi-area system under a conventional environment is considered under investigation. Each Area consists of a thermal^4^ gas^18^ wind turbine generator (WTG)^25^ and a dish Stirling solar thermal^22^ (DSTS) plant. The capacity ratio of Area 1: Area 2 = 1:2. The thermal units are equipped with a reheat turbine, a generation rate constraint of 3%/min, and a governor dead band of 0.0036 Hz. Each generation in gas and thermal in individual areas contributes 50% specified as area participation factor apf_11_ = apf_12_ = apf_21_ = apf_22_ = 0.5. The area participation factor, by definition, states how much participation of the generation should have in a control area. It can also be stated that the proposed controller dispatches 50% of the product of the controller transfer function and the area control error for each generation in each control area. The system experiencing a 1% step load disturbance exhibits combined voltage and frequency control and hence can be explained in the ALFC and AVR loops. The ALFC loop consists of the power generation by each source, where active power control through frequency adjustment is taken care of. The AVR loop consists of the exciter circuit and the amplifier for the generators. The above combination constitutes the nominal model. Further, an AC/direct current parallel link is also connected in both the control areas to create an AC/direct current bus for the proposed system. The transfer function modeling of the proposed Power system model is shown in Figure 1.

 The nominal parameters of Thermal, gas, WTG and DSTS sources are considered from Ref. 4, Ref. 18, Ref. 25, and Ref. 22 respectively. The nominal parameters of the AVR loop, i.e., exciter, amplifier, etc., are considered from Ref. 4 and Ref. 5. The discussion on the cross-coupling effect is explained in the next Sect. 3.1. The modeling of an electric spring as a voltage compensator is introduced in Sect. 3.2.

### Cross-coupling effect and ac/direct current parallel link

**Fig. 2:**
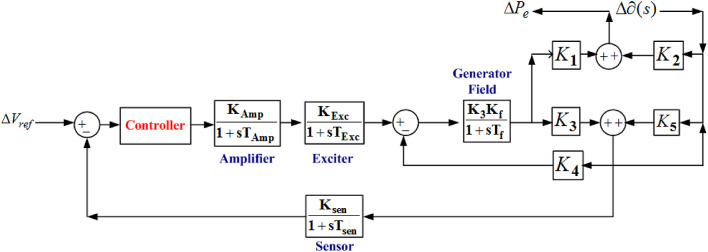
Transfer function model of the AVR system.

When two control areas are interconnected, load disturbances in one area impact another control area regarding frequency and tie-power deviation. However, when the automatic voltage control (AVR) loop is considered, a cross-coupling between the ALFC and the AVR loop exists them. A slight change in terminal voltage in the AVR loop will impact the frequency or speed of the generator in the other control area. However, upon load disturbances in one area, speed or frequency change in one area may not have a dominant effect on the terminal voltage of the generator. Thus, it is said that the cross-coupling is weak, but it exists and cannot be neglected. Hence, there is a requirement to observe the changes in the terminal generator due to load changes. The transfer function modeling of the AVR system in both the control areas are depicted in Figure 2.

The relationship between induced emf of synchronous generator stator depends on rotor speed and field current given by Eq. (1).

1 $$\:E\propto\:N\times\:{I}_{f}$$

Synchronous generator injects power to the grid as follows (2):

2 $$\:{P}_{e}=\frac{\left|V\right|\left|E\right|\text{s}\text{i}\text{n}\left(\delta\:\right)}{{X}_{s}}$$

Small disturbances in real power followed by the terminal voltage across the generator are given by Eqs. (3) and (4).

3 $$\:\varDelta\:{P}_{e}={P}_{s}\varDelta\:\partial\:+\:{K}_{2}\varDelta\:E$$

4 $$\:\varDelta\:V={K}_{5}\varDelta\:\partial\:+{K}_{6}\varDelta\:E$$

Where, P_s_, K_2_, K_5_¸and K_6_ represents the coefficients for depicting the cross-coupling effect. In the present scenario, only AC tie-line is interconnected. To model an AC/DC bus, presence of both AC and DC parallel links are necessary. Hence, a parallel DC link is connected which is operated in constant current control mode. The power flow in DC link depends on frequency deviation of the interconnected two areas as given by Eq. (5).

5 $$\:\varDelta\:{P}_{DC}\left(s\right)=\:\frac{{K}_{DC}}{1+s{T}_{DC}}\left[{\varDelta\:F}_{1}\left(s\right)-\:{\varDelta\:F}_{2}\left(s\right)\right]$$

Next section will demonstrate the modelling of an electric spring with special emphasis on the relation between the grid frequency and the DC input voltage.

### Electric spring as a voltage compensator

**Fig. 3:**
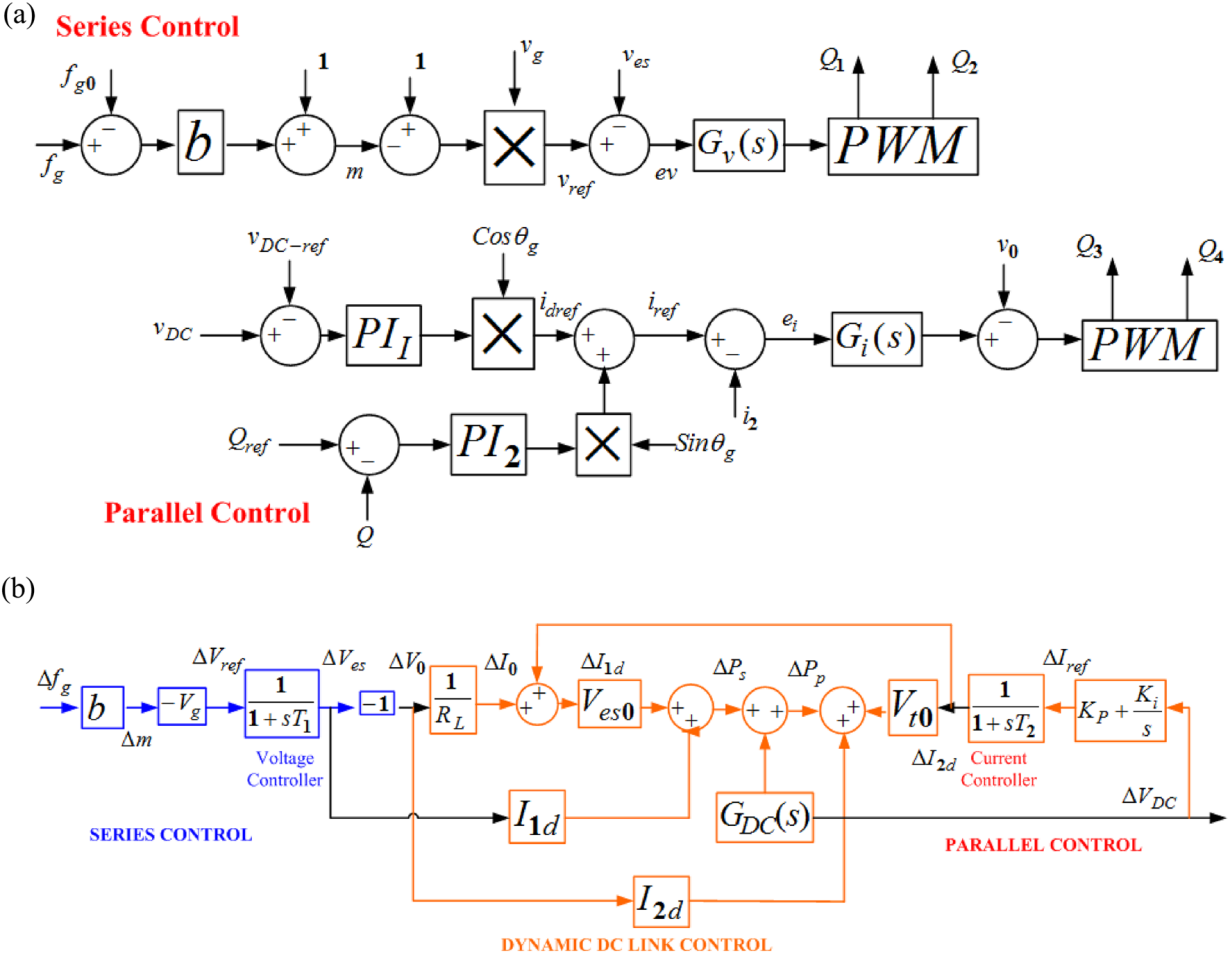
Schematic diagram of electric spring and its transfer function modeling form.

Electric Spring denotes a special form of reactive power controller. An electric spring can be used as a shock absorber or damper to mitigate voltage fluctuations. The schematic control structure can be visualized into series and parallel control. The schematic representation of series and parallel control of an electric spring is shown in FIGURE 3 (a). With reference to the FIGURE. 3(a), the series and parallel control can be mathematically established regarding grid frequency, DC voltage, load etc.

Electric Spring denotes a special form of reactive power controller. An electric spring can be used as a shock absorber or damper to mitigate voltage fluctuations. The schematic control structure can be visualized into series and parallel control. The schematic representation of series and parallel control of an electric spring is shown in FIGURE 3 (a). With reference to the FIGURE. 3(a), the series and parallel control can be mathematically established regarding grid frequency, DC voltage, load etc.

#### Series control

The series control controls the voltage V_O_ and regulates the grid frequency control. $\:{f}_{g}$ denotes the frequency lock loop. $\:{f}_{g0}$ is the nominal grid frequency, $\:{V}_{es}$ is the output voltage of the series converter^7^.

6 $$\:m=1+b.\left({f}_{g}-{f}_{g0}\right)=1-\frac{{V}_{es}}{{V}_{g}}$$

7 $$\:b=\:\frac{\varDelta\:{m}_{max}}{\varDelta\:{f}_{gmax}}$$

b is the proportional gain. $\:\varDelta\:{m}_{max}$ and $\:\varDelta\:{f}_{gmax}$ is the ratio of deviation and frequency deviation, respectively. To reduce the voltage tracking error, an inner loop controller is designed whose transfer function is given as: -

8 $$\:{G}_{v}\left(s\right)={k}_{pv}+\frac{{k}_{iv}\left(s\right)}{{s}^{2}+{f}_{g}^{2}}$$

$\:{k}_{pv}$ = proportional gain and $\:{k}_{pv}$ = resonant gain respectively.

#### Parallel control

The parallel control represents a current source. Another proportional-resonant controller is utilised given by Eq. (9). The orthogonal components i_dref_ and i_qref_ generates the reference current. DC Link voltage regulates the magnitude of i_dref_ reactive power regulates the magnitude of i_qref_. Q_ref_ is considered as zero so that the electric spring operates with a unity power factor.

9 $$\:{G}_{i}\left(s\right)={k}_{pi}+\frac{{k}_{ii}\left(s\right)}{{s}^{2}+{f}_{g}^{2}}$$

$$\:\left\{\begin{array}{c}\varDelta\:{P}_{s}={V}_{es0}.\varDelta\:{I}_{1d}+{I}_{1d0}.{\varDelta\:V}_{es}\\\:\varDelta\:{P}_{s}=\frac{{R}_{L}b{V}_{g}{I}_{Id0}-b{V}_{g}{V}_{es0}}{{R}_{L}\left(1+s{\tau\:}_{1}\right)}\varDelta\:{f}_{g}+{V}_{es0}\frac{{G}_{P1}\left(s\right)}{(1+s{\tau\:}_{2})}\varDelta\:{V}_{dc}\end{array}\right\}$$

$$\:\left\{\begin{array}{c}\varDelta\:{P}_{P}={V}_{o0}.\varDelta\:{I}_{2d}+{I}_{2d0}.{\varDelta\:V}_{0}\\\:\varDelta\:{P}_{P}=\frac{{V}_{o0}.{G}_{P1}\left(s\right)}{(1+s{\tau\:}_{2})}\varDelta\:{V}_{dc}-\frac{b{V}_{g}{I}_{2d0}}{\left(1+s{\tau\:}_{1}\right)}\varDelta\:{f}_{g}\end{array}\right\}$$

10 $$\:\varDelta\:{P}_{s}+\varDelta\:{P}_{d}={G}_{DC}\left(s\right).\varDelta\:{V}_{dc}=\frac{1}{{C}_{dc}{V}_{dc}s}\varDelta\:{V}_{dc}$$

11 $$\:\frac{\varDelta\:{V}_{dc}}{\varDelta\:{f}_{g}}=\frac{\frac{\left({b}^{2}{V}_{g}^{2}-b{V}_{g}{V}_{es0}\right)}{{R}_{L}\left(1+s{\tau\:}_{1}\right)}}{{G}_{dc}\left(s\right)-\frac{{V}_{g}{G}_{PI1}\left(s\right)}{\left(1+s{\tau\:}_{2}\right)}}$$

From FIGURE 3(b), $\:{\varDelta\:f}_{g}$ is the output of the ALFC loop, considered the frequency deviation, which is connected to the AVR loop and AC/DC link with the forward path of the tie-line. $\:{\varDelta\:V}_{DC}$ is the required output of the electric spring, which reduces the voltage deviation, if any, during the load fluctuations. The model in FIGURE 3(b) represents the implementation of an electric spring in a first order linearised system. The gains and time constants of the parameters and variables used in the equations can be found in Ref. 7–9, 10–16.

The nominal parameters of the series and parallel converter of the electric spring are tabulated in Table 1. These parameters are linearized to obtain the transfer function of ES, as in FIGURE 3(b).

**Table 1:** Nominal parameters for the modeling of electric spring.

Parameters	Description	Values
$\:{V}_{g}$	Magnitude of AC voltage	110 V
$\:{f}_{g}$	Frequency of grid	60 Hz
$\:{V}_{dc}$	DC-input voltage	400 V
$\:{C}_{dc\text{1,2}}$	DC-link capacitors	940 µF
$\:{X}_{f\text{1,2}}$	Filter inductors	2 mH
$\:{C}_{f1}$	Filter capacitor	15 µF
$\:{R}_{L}$	NCL resistance	20 Ω
$\:{f}_{sw}$	Switching frequency	20 kHz
$\:{k}_{pv},\:{k}_{pi}$	Proportional gains in PR	0.1, 0.1
$\:{k}_{iv},\:{k}_{ii}$	Resonant gains in PR	100, 50
$\:{k}_{p1}$	Proportional gain in PI	0.2
$\:{k}_{i1}$	Resonant gain in PI	5
$\:b$	Conversion ratio	0.15

## Problem formulation

Coordinated voltage and frequency control is to be studied in a multi-source environment. Presence of AC and DC bus is modelled as a parallel HVDC link. Further, electric spring is modelled along with the parallel AC/DC tie – line under conventional environment. A cascade controller 2DOFPI – PID controller is utilized in both control areas for ensuring the area control error to be zero. The objective function considered for the problem is Integral time multiplied by absolute error (ITAE) which is given by Eq. (12).

12 $$\:{J}_{ITAE}=\int\:\left(\varDelta\:{F}_{1}+\:\varDelta\:{F}_{2}+\varDelta\:{P}_{tie12}\right).t$$

### Cascade Controller and 2 DOF controller structure

**Fig. 4:**
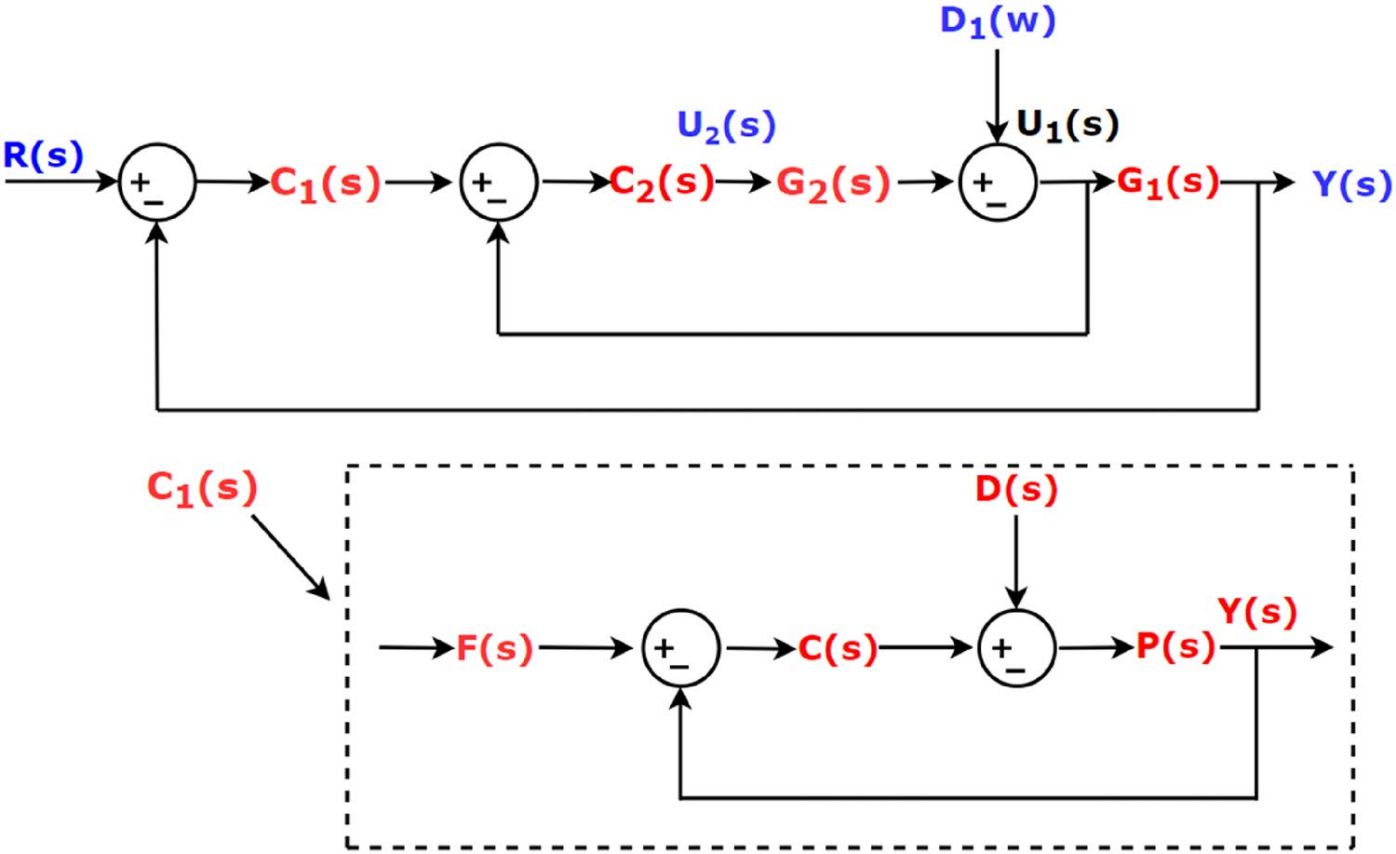
Control structure of the cascade controller along with control structure of a 2 DOF controller.

The choice of cascade controller^27^ in a system allows faster rejection of disturbances even before it propagates to other parts of the power system. A cascade controller in its simplest form has two loops – Outer Loop and Inner Loop as shown in FIGURE. 4.

#### Outer loop

The outer loop is known as the master loop which is given by Eq. (13).

13 $$\:Y\left(s\right)={G}_{1}\left(s\right){U}_{1}\left(s\right)+{d}_{1}\left(s\right)$$

where the input is $\:{U}_{1}\left(s\right)=\:{y}_{2}\left(s\right).\:\:\text{T}\text{h}$e inner process output $\:{y}_{2}\left(s\right)\:$becomes the input $\:{U}_{1}\left(s\right)\:$to the outer process.

#### Inner loop

The inner loop is known as the slave loop. G_2_(s) represents the process of inner loop. The equation of inner loop is given as.

14 $$\:{y}_{2}\left(s\right)={G}_{2}\left(s\right){U}_{2}\left(s\right)$$

where $\:{U}_{2}\left(s\right)$ = the inner process input. Similarly, $\:{U}_{1}\left(s\right)=\:{y}_{2}\left(s\right)$.

In the cascade controller, disturbances in inner loop does not reflect on the outer loop. There are provisions of corrections as inner loop is faster than the outer loop. Referring to sub figure in FIGURE 4, the transfer function of the 2DOF controller structure is given by Eqs. (15) and (16).

15 $$\:F\left(s\right)=\:\frac{\left(b{K}_{P}+cW{K}_{D}\right){s}^{2}+\left(b{K}_{P}N+c{K}_{I}\right)s+{K}_{I}N}{\left({K}_{P}+{K}_{D}N\right){s}^{2}+\left({K}_{P}N+{K}_{I}\right)s+{K}_{I}N}$$

16 $$\:C\left(s\right)=\frac{\left({K}_{P}+{K}_{D}N\right){s}^{2}+\left({K}_{P}N+{K}_{I}\right)s+{K}_{I}N}{s(s+N)}$$

Where the controller gains in their bounds are specified as follows: -

17 $$\:\left\{\begin{array}{c}{K}_{P-DOFmax}^{i}<{K}_{P-DOF}^{i}<{K}_{P-DOFmin}^{i}\\\:\begin{array}{c}{K}_{I-DOFmax}^{i}<{K}_{I-DOF}^{i}<{K}_{I-DOFmin}^{i}\\\:\begin{array}{c}{K}_{D-DOFmax}^{i}<{K}_{D-DOF}^{i}<{K}_{D-DOFmin}^{i}\\\:{N}_{DOFmax}^{i}<{N}_{DOF}^{i}<{N}_{DOFmin}^{i}\end{array}\\\:{b}_{DOFmax}^{i}<{b}_{DOF}^{i}<{b}_{DOFmin}^{i}\end{array}\\\:\begin{array}{c}{c}_{DOFmax}^{i}<{c}_{DOF}^{i}<{c}_{DOFmin}^{i}\\\:{K}_{P-PIDmax}^{i}<{K}_{P-PID}^{i}<{K}_{P-PIDmin}^{i}\\\:\begin{array}{c}{K}_{I-PIDmax}^{i}<{K}_{I-PID}^{i}<{K}_{I-PIDmin}^{i}\\\:{K}_{D-PIDmax}^{i}<{K}_{D-PID}^{i}<{K}_{D-PIDmin}^{i}\\\:{N}_{PIDmax}^{i}<{N}_{PID}^{i}<{N}_{PIDmin}^{i}\end{array}\end{array}\end{array}\right\}$$

The controller gains $\:{K}_{P-DOF}^{i}$, $\:{K}_{I-DOF}^{i}$, $\:{K}_{D-DOF}^{i},\:\:{K}_{P-PID}^{i},\:{K}_{I-PID}^{i}$, $\:{K}_{D-PID}^{i}\:$specified with lower bounds as 0 and the upper bounds as 1. The weighted co-efficient of degree of freedom controllers $\:{b}_{DOF}^{i}\:$and $\:{c}_{DOF}^{i}\:$ranges from 1 to 5. The filter co-efficient $\:{N}_{DOF}^{i}$ and $\:{N}_{PID}^{i}$ ranges from 1 to 100. Golden Jackal Optimization technique is utilized to tune the controller gains, weighted coefficients, and filter co-efficient of the proposed 2DOF – PID Controller.

## Golden Jackal optimization

Nitish Chopra and Mohsin Ansari proposed the powerful golden jackal optimization (GJO) in the year 2022. GJO mimics how the golden jackal hunts^28– 29^. Both make and female jackals participate in hunting. Golden Jackals are of moderate size and are terrestrial predators of canine family. They are of small size but have long legs which help them to run for long distances to hunt for its prey. The hunting behaviour can be explained in three steps: -.

Step 1: Exploration of the prey.

Step 2: Prey Surrounding so that it cannot run and then disturbing it until it tries to move.

Step 3: Attacking the prey with a jump when it moves or tries to escape.

**Search Space**: GJO is a flock - based method where the first term is randomly generated from the search space as the first trail value. The first trail value is calculated as follows: -

18 $$\:{Y}_{0}={Y}_{min}+rand\left({Y}_{max}-{Y}_{min}\right)$$

where, Y_max_ and Y_min_ correspond to the bounds i.e. lower/upper from search space, respectively and rand denotes a uniform random number that lies inside [0,1]. The first position matrix of the prey is provided in Eq. (19).

19 $$\:Prey=\left[\begin{array}{c}{Y}_{\text{1,1}}\:{Y}_{\text{1,2}\:}{........Y}_{1,d}\\\:{Y}_{\text{2,1}}\:{Y}_{\text{2,2}\:}{........Y}_{2,d}\\\:{Y}_{n,1}\:{Y}_{n,2\:}{........Y}_{n,d}\end{array}\right]$$

where, Y_i, j_ is the j_th_ dimension of i_th_ prey. n represents total number of prey and d represents the dimension. Prey positions are the parameters of a particular position. Fitness value represents the objective function throughout the optimization phase. The fitness value can be represented as:

20 $$\:{F}_{O}=\left[\begin{array}{c}f\left({Y}_{\text{1,1}}\:{Y}_{\text{1,2}\:}{........Y}_{1,d}\right)\\\:f\left({Y}_{\text{2,1}}\:{Y}_{\text{2,2}\:}{........Y}_{2,d}\right)\\\:f\left({Y}_{n,1}\:{Y}_{n,2\:}{........Y}_{n,d}\right)\end{array}\right]$$.

where, **F**_**O**_ contains all fitness value of prey and **f** denotes the fitness function or performance index. The best fit value corresponds to a male jackal, while the second best represents a female jackal.

**Exploration**: All golden jackals need to detect their prey. However, prey shall run away from being captured. Then, male jackals will hunt for new prey and the female trails the male jackal as follows: -

21 $$\:\left(\begin{array}{c}{Y}_{1}\left(t\right)\:={Y}_{M}\left(t\right)\:-\:E.\left|{Y}_{M}\left(t\right)-rl.Prey\left(t\right)\right|\\\:{Y}_{2}\left(t\right)\:={Y}_{FM}\left(t\right)\:-\:E.\left|{Y}_{FM}\left(t\right)-rl.Prey\left(t\right)\right|\end{array}\right)$$

where, t:- current iterations, prey(t) :- present position, and Y_M_ (t) and Y_FM_ (t):- the male and female jackal position in the search space, respectively. E:- escape energy of prey and is depicted as follows: -

22 $$\:E={E}_{1}\times\:{E}_{0}$$

where, E_0,_ E_1_ :- initial energy state and decreasing energy state of the prey.

23 $$\:{E}_{0}=(2\times\:r)-1$$

24 $$\:{E}_{1}={C}_{1}\times\:(1-^{t}/T)$$

t and T represent the current and maximum iteration, respectively. E_1_ range: 1.5–0. The r_l_ represents the arbitrary random vector based on the levy flight (LF) distribution and is calculated as:

25 $$\:rl=0.05\times\:LF\left(y\right)$$

The LF is calculated by using Eq. (30)

26 $$\:LF(y)=\frac{0.01\times\:(\mu\:\times\:\sigma\:)}{\left|{v}^{(1/ \beta)}\right|}$$

27 $$\:\sigma\:={\left(\frac{{\Gamma\:}\left(1+\beta\:\right)\times\:Sin\left(\frac{\pi\:\beta\:}{2}\right)}{{\Gamma\:}\left(\frac{1+\beta\:}{2}\right)\times\:\beta\:\times\:{2}^{\left(\frac{\beta\:-1}{2}\right)}}\right)}^{\raisebox{1ex}{$1$}\!\left/\:\!\raisebox{-1ex}{$\beta\:$}\right.}$$

where, µ, σ are numbers between 0 and 1, and β = 1.5. Finally, golden jackal’s updated position is:

28 $$\:Y\left(t+1\right)=\frac{{Y}_{1}\left(t\right)+{Y}_{2}\left(t\right)}{2}$$

### Exploitation

Exploitation phase refers to the encirclement of the prey by the jackals and locating them. After the encirclement, jackals jump on the prey and eat them. Also, exploitation phase reduces the chances of escape of its prey. The hunting phenomenon expressing the male and female jackal, is given as below.

29 $$\:\left(\begin{array}{c}{Y}_{1}\left(t\right)={Y}_{M}\left(t\right)-E.\left|rl.{Y}_{M}\left(t\right)-Prey\left(t\right)\right|\\\:{Y}_{2}\left(t\right)={Y}_{FM}\left(t\right)-E.\left|rl.{Y}_{FM}\left(t\right)-Prey\left(t\right)\right|\end{array}\right)$$

***rl*** in Eqs. (32) and (33) introduces randomness in the exploitation period, highlighting large search area and avoiding local optimum.

## Results and analysis

### Case study 1 selection of secondary ALFC and AVR controller in the two-area power system

This case study is carried out to determine the (i) best secondary controller for the frequency control and (ii) best controller for the voltage control. The proposed system consists of two areas; hence, there are two controllers each for frequency (ALFC) and voltage control (AVR). Three types of frequency and voltage controllers, such as proportional–-integral–derivative (PID), two degrees of freedom proportional–integral–-integral-derivative (2DOF PID) and two degrees of freedom proportional–integral–derivative cascaded with proportional–integral–derivative controller (2DOF PID – PID) are compared for performance evaluation. A powerful Golden Jackal Optimization tunes the controller gains and parameters. The controllers are utilized one at a time and the optimum controller gains are tabulated in Table 2.

**Table 2:** Optimum controller gains and parameters for PID, 2DOF PID and 2DOF PID – PID controllers.

Controllers	Optimum gains	PID	2DOF PID	2DOF PID - PID
Inner loop ALFC Controller 1	$\:{\text{K}}_{\text{P}1}^{\text{*}}$	0.8971	0.9123	0.9986
	$\:{\text{K}}_{\text{I}1}^{\text{*}}$	0.9087	0.0978	0.0789
	$\:{\text{K}}_{\text{D}1}^{\text{*}}$	0.8876	0.7856	0.9087
	$\:{\text{N}}_{1}^{\text{*}}$	99.087	79.219	88.987
	$\:{\text{b}}_{1}^{\text{*}}$	3.4597	2.1345	3.7651
	$\:{\text{c}}_{1}^{\text{*}}$	4.8907	3.2347	2.8790
Inner loop AVR Controller 1	$\:{\text{K}}_{\text{P}2}^{\text{*}}$	0.9967	0.8970	0.9870
	$\:{\text{K}}_{\text{I}2}^{\text{*}}$	0.0986	0.0879	0.0678
	$\:{\text{K}}_{\text{D}2}^{\text{*}}$	0.8956	0.9897	0.8907
	$\:{\text{N}}_{2}^{\text{*}}$	0.9807	89.897	0.6785
	$\:{\text{b}}_{2}^{\text{*}}$	3.4567	4.6753	4.7865
	$\:{\text{c}}_{2}^{\text{*}}$	2.4567	3.5673	3.8907
Inner loop ALFC Controller 2	$\:{\text{K}}_{\text{P}3}^{\text{*}}$	0.8976	0.9086	0.8976
	$\:{\text{K}}_{\text{I}3}^{\text{*}}$	0.7896	0.9897	0.7896
	$\:{\text{K}}_{\text{D}3}^{\text{*}}$	0.9876	0.8870	0.9087
	$\:{\text{N}}_{3}^{\text{*}}$	89.098	97.894	89.786
	$\:{\text{b}}_{3}^{\text{*}}$	3.5674	2.4567	0.8906
	$\:{\text{c}}_{3}^{\text{*}}$	3.6784	4.8967	3.6781
Inner loop AVR Controller 2	$\:{\text{K}}_{\text{P}4}^{\text{*}}$	0.8977	0.6793	0.7890
	$\:{\text{K}}_{\text{I}4}^{\text{*}}$	0.0987	0.0985	0.0978
	$\:{\text{K}}_{\text{D}4}^{\text{*}}$	0.9807	0.8978	0.9078
	$\:{\text{N}}_{4}^{\text{*}}$	90.897	98.784	99.235
	$\:{\text{b}}_{4}^{\text{*}}$	3.5679	2.4567	3.6789
	$\:{\text{c}}_{4}^{\text{*}}$	4.5672	4.7682	2.8907
Outer loop ALFC Controller 1	$\:{\text{K}}_{\text{P}5}^{\text{*}}$	0.9876	0.8976	0.9986
	$\:{\text{K}}_{\text{I}5}^{\text{*}}$	0.0989	0.0876	0.0678
	$\:{\text{K}}_{\text{D}5}^{\text{*}}$	0.7895	0.4689	0.7896
	$\:{\text{N}}_{5}^{\text{*}}$	78.906	66.890	89.789
Outer loop AVR Controller 1	$\:{\text{K}}_{\text{P}6}^{\text{*}}$	0.9807	0.3467	0.6783
	$\:{\text{K}}_{\text{I}6}^{\text{*}}$	0.9087	0.6758	0.7856
	$\:{\text{K}}_{\text{D}6}^{\text{*}}$	0.9086	0.8895	0.9803
	$\:{\text{N}}_{6}^{\text{*}}$	90.567	49.097	89.789
Outer loop ALFC Controller 2	$\:{\text{K}}_{\text{P}7}^{\text{*}}$	0.9876	0.7657	0.8790
	$\:{\text{K}}_{\text{I}7}^{\text{*}}$	0.0987	0.8976	0.9087
	$\:{\text{K}}_{\text{D}7}^{\text{*}}$	0.9098	0.9078	0.8879
	$\:{\text{N}}_{7}^{\text{*}}$	89.097	56.789	89.456
Outer loop AVR Controller 2	$\:{\text{K}}_{\text{P}8}^{\text{*}}$	0.9087	0.8970	0.9786
	$\:{\text{K}}_{\text{I}8}^{\text{*}}$	0.8976	0.0567	0.9086
	$\:{\text{K}}_{\text{D}8}^{\text{*}}$	0.7786	0.8756	0.9986
	$\:{\text{N}}_{8}^{\text{*}}$	78.674	88.890	90.087

**Fig. 5:**
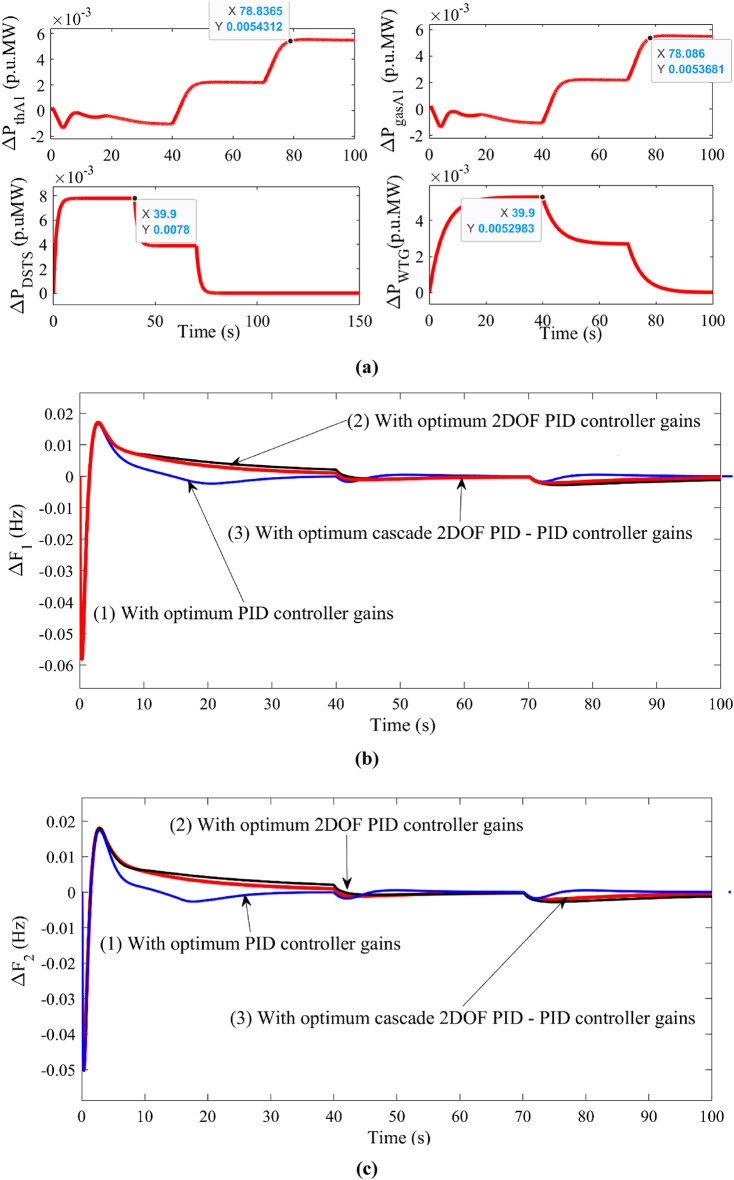

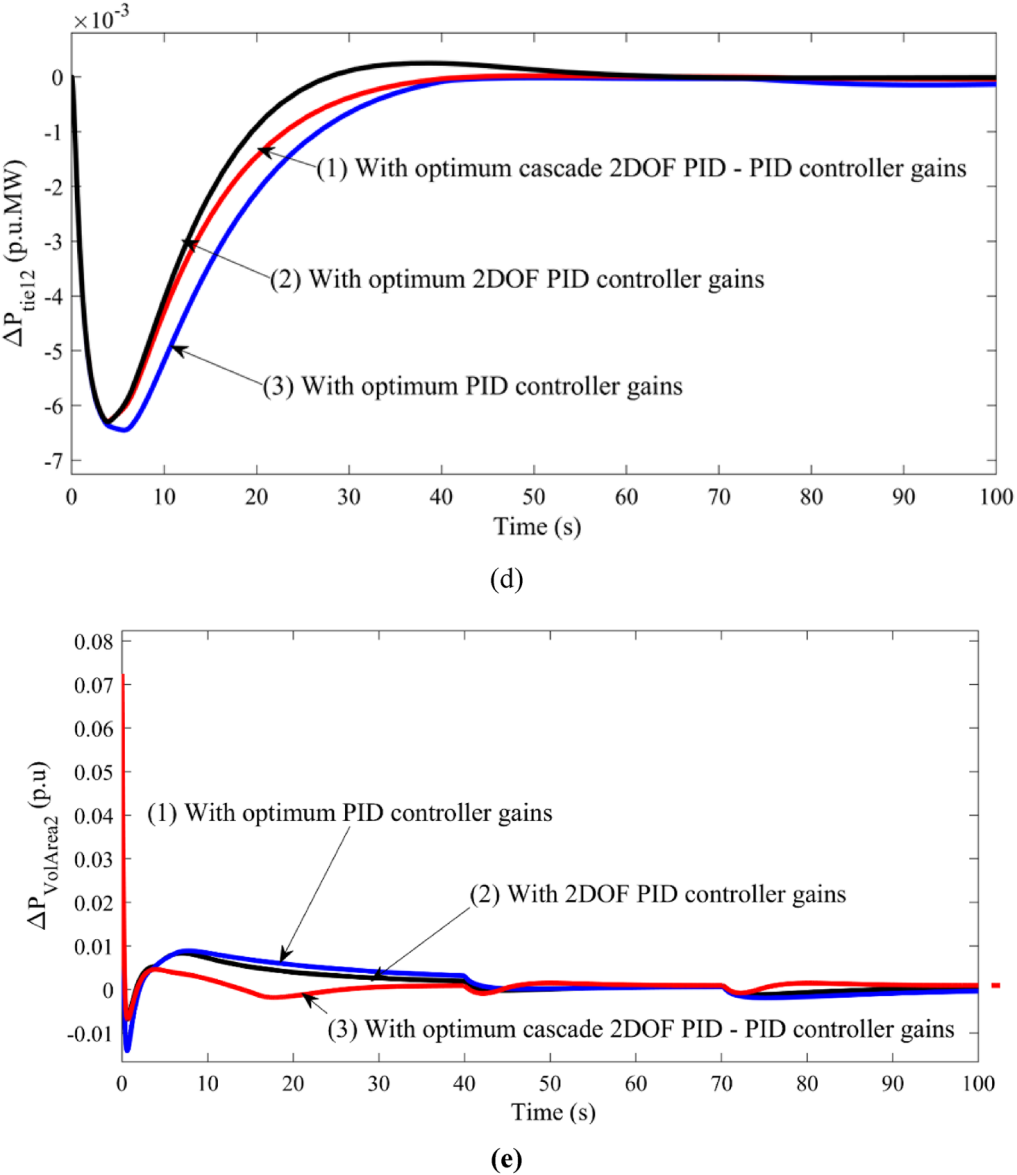
Comparison of system dynamic responses for different types of controllers in the proposed system such as PID, 2 DOF PID, 2DOF PID – PID controllers.

- $$\:\varDelta\:{\text{P}}_{\text{g}}\left(\text{t}\right)\:\text{v}\text{s}\:\text{t}\text{i}\text{m}\text{e}$$ 
- $$\:\varDelta\:{\text{F}}_{1}\left(\text{t}\right)\:\text{v}\text{s}\:\text{t}\text{i}\text{m}\text{e}$$ 
- $$\:\varDelta\:{\text{F}}_{2}\left(\text{t}\right)\:\text{v}\text{s}\:\text{t}\text{i}\text{m}\text{e}$$ 
- $$\:\varDelta\:{\text{P}}_{\text{t}\text{i}\text{e}12}\left(\text{t}\right)\:\text{v}\text{s}\:\text{t}\text{i}\text{m}\text{e}$$ 
- $$\:\varDelta\:{\text{P}}_{\text{V}\text{o}\text{l}\text{A}\text{r}\text{e}\text{a}2}\left(\text{t}\right)\:\text{v}\text{s}\:\text{t}\text{i}\text{m}\text{e}$$ 

From the system responses shown in FIGURE 5, the cascade controller 2DOF PID – PID successfully reduces the frequency deviation, generator terminal voltage and tie-power deviation to zero and proves to be better than the other controllers. The difference in overshoot for the controllers may seem less because the load disturbance is 1%. However, the settling time due to the proposed cascade controller outperforms others.

### Case study 2 impact of various performance index on the proposed system

From the previous investigation, it is evident that the cascade controller 2DOF PID - PID successfully mitigates the area control error to zero. In this case study, it is necessary to determine the best performance index among the classical indices such as Integral of Squared error (ISE), Integral of absolute error (IAE), Integral of time multiplied by squared error (ITSE), and Integral of time multiplied by absolute error (IAE). In each case, using the indices one at a time, the proposed cascade controller 2DOF PID – PID is utilized, whose controller gains are tuned by the Golden Jackal Optimization technique. The tuned parameters of the cascade 2DOF PID – PID controller are tabulated in Table 3.

**Table 3:** Optimum controller gains of the proposed cascade controller.

Controllers	Optimum gains	ISE	IAE	ITSE	ITAE
Inner loop ALFC Controller 1	$\:{\text{K}}_{\text{P}1}^{\text{*}}$	0.6795	0.8796	0.9786	0.6754
	$\:{\text{K}}_{\text{I}1}^{\text{*}}$	0.0674	0.8876	0.8890	0.9085
	$\:{\text{K}}_{\text{D}1}^{\text{*}}$	0.9867	0.9087	0.9867	0.9876
	$\:{\text{N}}_{1}^{\text{*}}$	90.876	87.908	99.897	89.786
	$\:{\text{b}}_{1}^{\text{*}}$	2.5673	4.8976	4.4876	4.7863
	$\:{\text{c}}_{1}^{\text{*}}$	4.5673	3.9908	5.7860	3.6753
Inner loop AVR Controller 1	$\:{\text{K}}_{\text{P}2}^{\text{*}}$	0.9867	0.6789	0.7865	0.9876
	$\:{\text{K}}_{\text{I}2}^{\text{*}}$	0.8765	0.9087	0.9897	0.6753
	$\:{\text{K}}_{\text{D}2}^{\text{*}}$	0.8976	0.9981	0.8975	0.9076
	$\:{\text{N}}_{2}^{\text{*}}$	98.345	78.675	89.907	90.897
	$\:{\text{b}}_{2}^{\text{*}}$	4.5679	3.6754	4.9056	3.5623
	$\:{\text{c}}_{2}^{\text{*}}$	3.7676	4.3452	4.9087	4.7865
Inner loop ALFC Controller 2	$\:{\text{K}}_{\text{P}3}^{\text{*}}$	0.3324	0.8976	0.6754	0.8976
	$\:{\text{K}}_{\text{I}3}^{\text{*}}$	0.6734	0.2341	0.9086	0.8976
	$\:{\text{K}}_{\text{D}3}^{\text{*}}$	0.8976	0.9074	0.6754	0.9086
	$\:{\text{N}}_{3}^{\text{*}}$	77.675	88.749	89.907	90.893
	$\:{\text{b}}_{3}^{\text{*}}$	3.2345	4.6754	4.7865	4.7653
	$\:{\text{c}}_{3}^{\text{*}}$	4.5432	4.5643	3.7865	5.0978
Inner loop AVR Controller 2	$\:{\text{K}}_{\text{P}4}^{\text{*}}$	0.9876	0.8123	0.6754	0.8967
	$\:{\text{K}}_{\text{I}4}^{\text{*}}$	0.0879	0.0989	0.9086	0.9078
	$\:{\text{K}}_{\text{D}4}^{\text{*}}$	0.6789	0.7433	0.6758	0.0978
	$\:{\text{N}}_{4}^{\text{*}}$	98.768	78.890	89.097	99.876
	$\:{\text{b}}_{4}^{\text{*}}$	4.5648	3.4567	4.6754	3.4523
	$\:{\text{c}}_{4}^{\text{*}}$	3.7654	4.6754	5.7856	4.3674
Outer loop ALFC Controller 1	$\:{\text{K}}_{\text{P}5}^{\text{*}}$	0.8769	0.9087	0.8976	0.6342
	$\:{\text{K}}_{\text{I}5}^{\text{*}}$	0.0987	0.0878	0.0945	0.0878
	$\:{\text{K}}_{\text{D}5}^{\text{*}}$	0.8976	0.2318	0.6754	0.7863
	$\:{\text{N}}_{5}^{\text{*}}$	79.896	99.897	56.786	89.789
Outer loop AVR Controller 1	$\:{\text{K}}_{\text{P}6}^{\text{*}}$	0.8967	0.9087	0.9087	0.8976
	$\:{\text{K}}_{\text{I}6}^{\text{*}}$	0.0978	0.8976	0.8907	0.6743
	$\:{\text{K}}_{\text{D}6}^{\text{*}}$	0.8907	0.8979	0.6758	0.9056
	$\:{\text{N}}_{6}^{\text{*}}$	67.890	78.675	89.765	90.897
Outer loop ALFC Controller 2	$\:{\text{K}}_{\text{P}7}^{\text{*}}$	0.8965	0.9086	0.8907	0.6734
	$\:{\text{K}}_{\text{I}7}^{\text{*}}$	0.0899	0.0908	0.0675	0.8986
	$\:{\text{K}}_{\text{D}7}^{\text{*}}$	0.8976	0.9087	0.6754	0.9065
	$\:{\text{N}}_{7}^{\text{*}}$	79.906	87.675	89.890	67.546
Outer loop AVR Controller 2	$\:{\text{K}}_{\text{P}8}^{\text{*}}$	0.4564	0.8976	0.6754	0.7865
	$\:{\text{K}}_{\text{I}8}^{\text{*}}$	0.6784	0.9085	0.8979	0.5643
	$\:{\text{K}}_{\text{D}8}^{\text{*}}$	0.8967	0.5643	0.5643	0.7865
	$\:{\text{N}}_{8}^{\text{*}}$	78.896	89.096	67.786	98.673

**Fig. 6:**
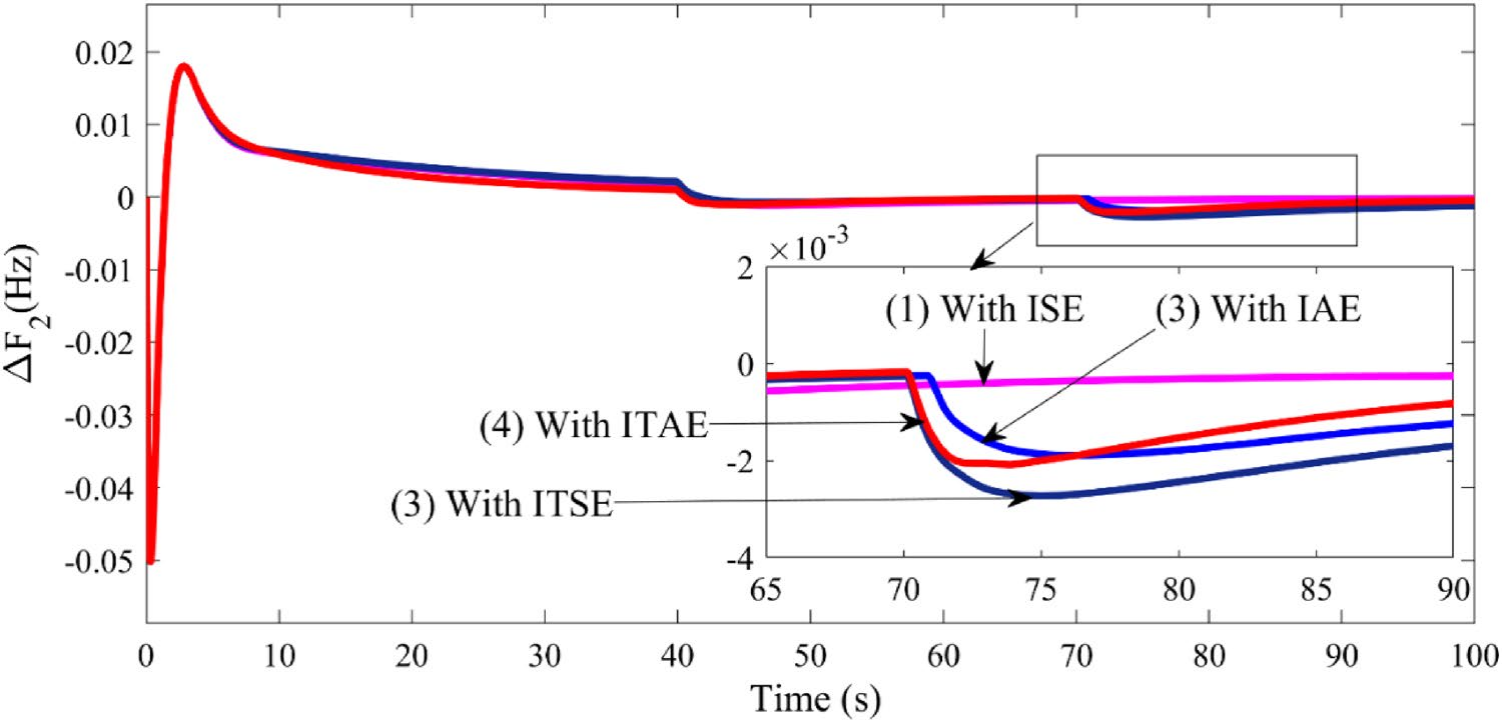
Comparison of frequency deviation in Area 2 vs. time for different performance indices.

The corresponding figure of frequency deviation in Area 2 vs. time is shown in Figure 6.

As the change in power generation is specifically shown at 40 s and 70 s for which the frequency deviation also witnesses changes. The differences in the performances due to all the indices are not that different but differ in terms of 0.008 on average. But the same difference is lesser in the case of ITAE as a performance index. Also, the value of the performance index ISE, IAE, ITSE, and ITAE are observed as 0.6789, 0.5492, 0.3298, and 0.1867, respectively. Thus, for further investigation, ITAE is considered the performance index.

### Case study 3 investigation with parallel AC/DC link on the proposed system

A study is carried out with parallel AC/DC link on the proposed two area hydro - thermal system coordinated with AVR. This case study is carried out to observe the system dynamic performance when there is a presence of parallel AC/DC link. The reason being that in a microgrid environment where various sources such as distributed energy sources, conventional and non-conventional sources, there is a requirement of both AC and DC bus followed by conversion as required. With consideration of ITAE as performance index, cascade controller is employed in both the control areas and the AVR systems and Golden Jackal Optimization is utilized in tuning the controller parameters. Technically, there should not be much change while implementing the parallel link. The optimal gains of all controller parameters tuned by GJO is tabulated in column 3 of Table 4.

**Table 4:** Optimum controller parameters tuned by GJO for different case studies.

	Optimum gains	Case study 3	Case study 4	Case study 5	Case study 6
Inner loop ALFC Controller 1	$\:{\text{K}}_{\text{P}1}^{\text{*}}$	0.9807	0.8898	0.6783	0.8879
	$\:{\text{K}}_{\text{I}1}^{\text{*}}$	0.0548	0.8909	0.0897	0.4521
	$\:{\text{K}}_{\text{D}1}^{\text{*}}$	0.8965	0.9089	0.9087	0,7867
	$\:{\text{N}}_{1}^{\text{*}}$	90.595	76.578	89.456	98.783
	$\:{\text{b}}_{1}^{\text{*}}$	4.6532	3.6542	3.2134	4.6532
	$\:{\text{c}}_{1}^{\text{*}}$	3.5647	4.3452	4.3242	2.8789
Inner loop AVR Controller 1	$\:{\text{K}}_{\text{P}2}^{\text{*}}$	0.8895	0.9989	0.8976	0.8798
	$\:{\text{K}}_{\text{I}2}^{\text{*}}$	0.8076	0.0767	0.9087	0.8076
	$\:{\text{K}}_{\text{D}2}^{\text{*}}$	0.7845	0.9045	0.8973	0.9870
	$\:{\text{N}}_{2}^{\text{*}}$	78.874	34.563	48.907	68.987
	$\:{\text{b}}_{2}^{\text{*}}$	2.3234	3.5643	4.9087	3.8965
	$\:{\text{c}}_{2}^{\text{*}}$	3.4532	7.6832	2.3423	4.8768
Inner loop ALFC Controller 2	$\:{\text{K}}_{\text{P}3}^{\text{*}}$	0.8934	0.9046	0.8976	0.9087
	$\:{\text{K}}_{\text{I}3}^{\text{*}}$	0.0897	0.0557	0.0980	0.8907
	$\:{\text{K}}_{\text{D}3}^{\text{*}}$	0.8763	0.7874	0.8935	0.6732
	$\:{\text{N}}_{3}^{\text{*}}$	90.483	80.987	89.893	90.897
	$\:{\text{b}}_{3}^{\text{*}}$	2.4532	3.2313	3.5674	4.3213
	$\:{\text{c}}_{3}^{\text{*}}$	4.6578	4.2312	3.2313	4.6732
Inner loop AVR Controller 2	$\:{\text{K}}_{\text{P}4}^{\text{*}}$	0.4387	0.8964	0.7863	0.8973
	$\:{\text{K}}_{\text{I}4}^{\text{*}}$	0.0765	0.0879	0.0983	0.0879
	$\:{\text{K}}_{\text{D}4}^{\text{*}}$	0.9087	0.8972	0.9084	0.9980
	$\:{\text{N}}_{4}^{\text{*}}$	90.456	89.783	88.783	45.879
	$\:{\text{b}}_{4}^{\text{*}}$	3.2345	2.3421	4.7822	3.8976
	$\:{\text{c}}_{4}^{\text{*}}$	4.3256	3.6542	3.7664	4.3242
Outer loop ALFC Controller 1	$\:{\text{K}}_{\text{P}5}^{\text{*}}$	0.8764	0.9087	0.8973	0.9089
	$\:{\text{K}}_{\text{I}5}^{\text{*}}$	0.0786	0.0245	0.0432	0.0989
	$\:{\text{K}}_{\text{D}5}^{\text{*}}$	0.8973	0.9034	0.8973	0.9808
	$\:{\text{N}}_{5}^{\text{*}}$	98.763	78.896	90.893	88.789
Outer loop AVR Controller 1	$\:{\text{K}}_{\text{P}6}^{\text{*}}$	0.2371	0.5632	0.7863	0.8978
	$\:{\text{K}}_{\text{I}6}^{\text{*}}$	0.0893	0.0986	0.0879	0.0989
	$\:{\text{K}}_{\text{D}6}^{\text{*}}$	0.9087	0.6732	0.7739	0.8978
	$\:{\text{N}}_{6}^{\text{*}}$	78.894	89.896	99.897	78.673
Outer loop ALFC Controller 2	$\:{\text{K}}_{\text{P}7}^{\text{*}}$	0.9086	0.8907	0.2132	0.6532
	$\:{\text{K}}_{\text{I}7}^{\text{*}}$	0.0897	0.9863	0.0453	0.9082
	$\:{\text{K}}_{\text{D}7}^{\text{*}}$	0.8945	0.3421	0.7832	0.3213
	$\:{\text{N}}_{7}^{\text{*}}$	88.764	56.342	66.453	78.672
Outer loop AVR Controller 2	$\:{\text{K}}_{\text{P}8}^{\text{*}}$	0.9870	0.5632	0.9830	0.8973
	$\:{\text{K}}_{\text{I}8}^{\text{*}}$	0.0654	0.8096	0.4673	0.9087
	$\:{\text{K}}_{\text{D}8}^{\text{*}}$	0.9089	0.8879	0.7832	0.8897
	$\:{\text{N}}_{8}^{\text{*}}$	87.908	98.769	99.637	78.673

**Fig. 7:**
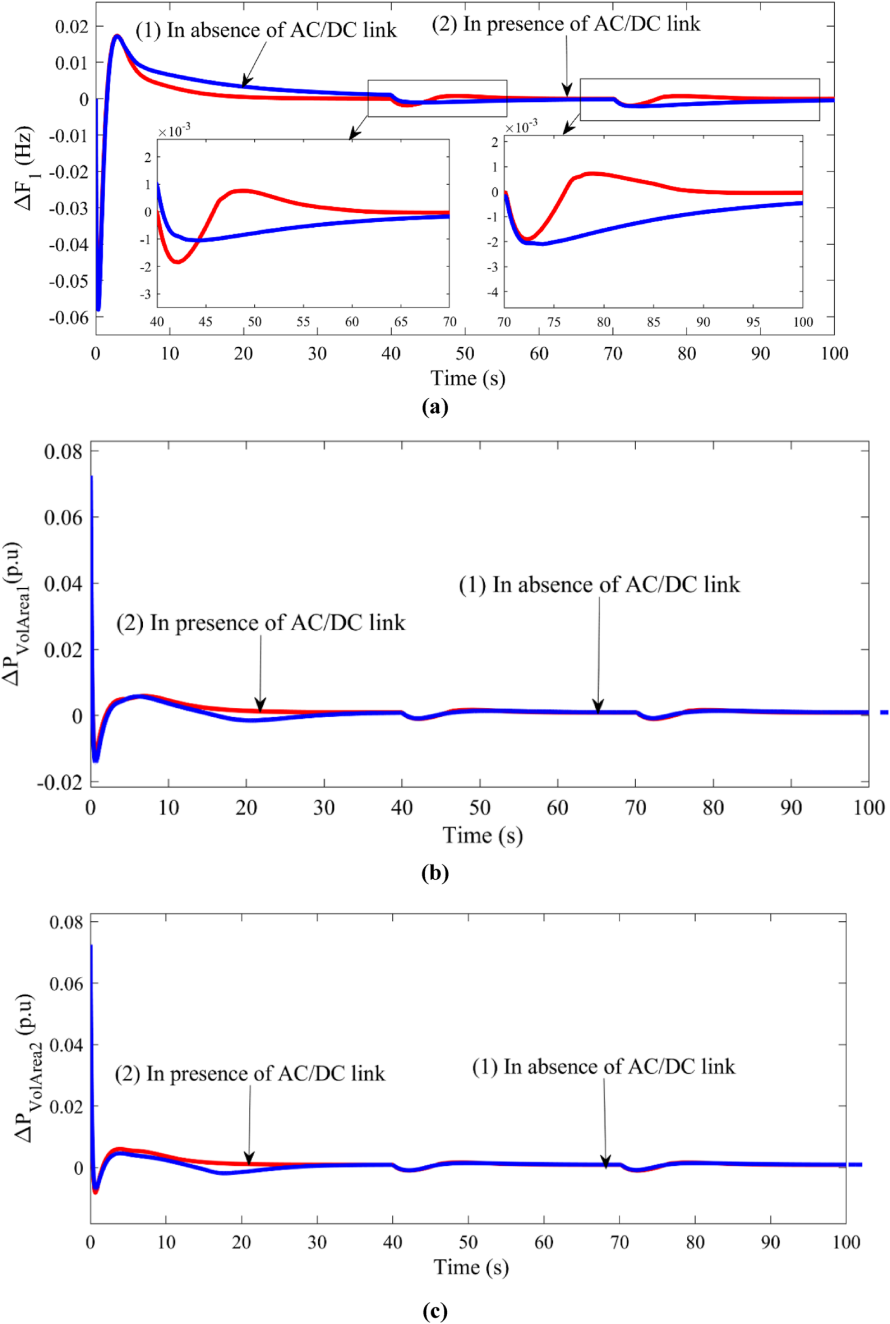
Comparison of system dynamic responses for AVR coordinated system in presence of parallel AC/DC link.

- ∆F_1_ (t) vs. time.
- ∆P_volArea1_ (t) vs. time.
- ∆P_volArea2_ (t) vs. time.

With the optimum parameters, system dynamic responses are plotted and compared with the responses with AC tie line only. FIGURE 7 ((a), (b) and (c)) depicts three figures: frequency deviation in Area 1 vs. time, Power deviation due to voltage in Area 1 vs. time, and Power deviation due to voltage in Area 2 vs. time. In each of the figures, it is observed that there is not much difference in the usage of AC and DC parallel links. However, parallel AC/DC link shows a slight response improvement.

### Case study **4** introducing electric spring and parallel ac/dc link in alfc avr coordinated proposed system

**Fig. 8:**
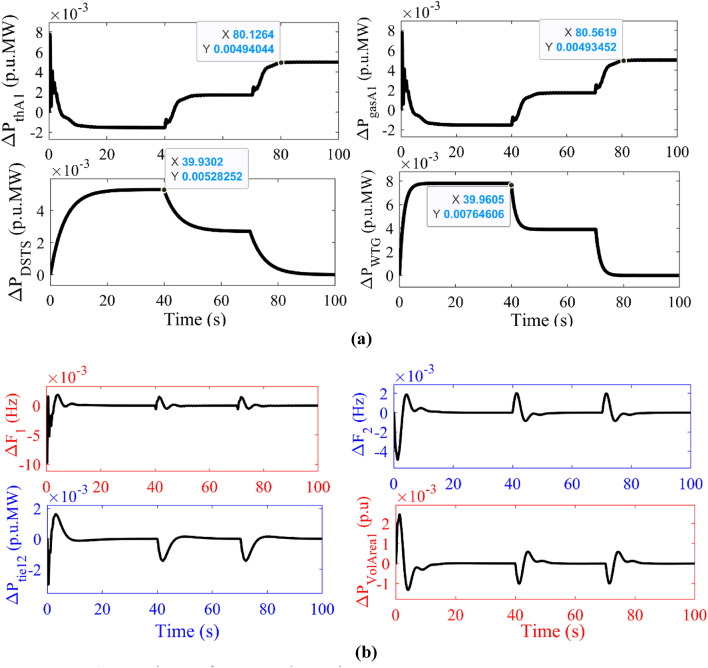
Comparison of system dynamic responses with presence of electric spring in the proposed system.

The concept of electric spring is modelled as shown in FIGURE 2 and integrated with parallel AC/DC link in the interconnected location of the two-area power system. Cascade controller is employed in all ALFC and AVR controllers tuned by GJO technique. The tuned parameters of the controller are tabulated in column 4 of Table 4. The corresponding system dynamic responses are plotted and depicted in FIGURE 8. FIGURE 8(a) depicts the power generation deviation vs. time in case of thermal plant, gas plant, DSTS and WTG in Area 1 respectively. FIGURE 8 (b) depicts the frequency deviation in Area 1 and Area 2, tie power deviation between Area 1 and Area 2, and Power deviation in Area 1 for voltage deviation in Area 1. Electric Spring is supposed to stabilize the voltage deviation in AVR, the change in voltage deviation is supposed to be made constant with the help of electric spring. The tuned controller parameters by GJO are tabulated in column 4 in Table 4.

- $$\:{\varDelta\:\text{P}}_{\text{t}\text{h}\text{A}1},\:{\varDelta\:\text{P}}_{\text{D}\text{S}\text{T}\text{S}},\:{\varDelta\:\text{P}}_{\text{g}\text{a}\text{s}\text{A}1\:},\:{\varDelta\:\text{P}}_{\text{W}\text{T}\text{G}}\:\text{v}\text{s}\:\text{t}\text{i}\text{m}\text{e}\:$$ 
- $$\:{\varDelta\:\text{F}}_{1},\:{\varDelta\:\text{F}}_{2},\:{{\varDelta\:\text{P}}_{\text{t}\text{i}\text{e}12}}_{\:},\:{\varDelta\:\text{P}}_{\text{v}\text{o}\text{l}\text{A}\text{r}\text{e}\text{a}1}\:\text{v}\text{s}\:\text{t}\text{i}\text{m}\text{e}$$ 

From all the figures (Figure 8) depicted, it is observed that electric spring can stabilise the power system instead of constant voltage fluctuations. Upon changes in terminal voltage of the generator, the fluctuations are taken care of by the proposed cascade controller. The overshoot and undershoot value of the response in power deviation in Area 2 due to terminal voltage changes is of satisfactory value and is reduced by 10% than with only ALFC - AVR coordination.

### Case study 5 investigating the proposed system and proposed controller with random loading pattern

**Fig. 9:**
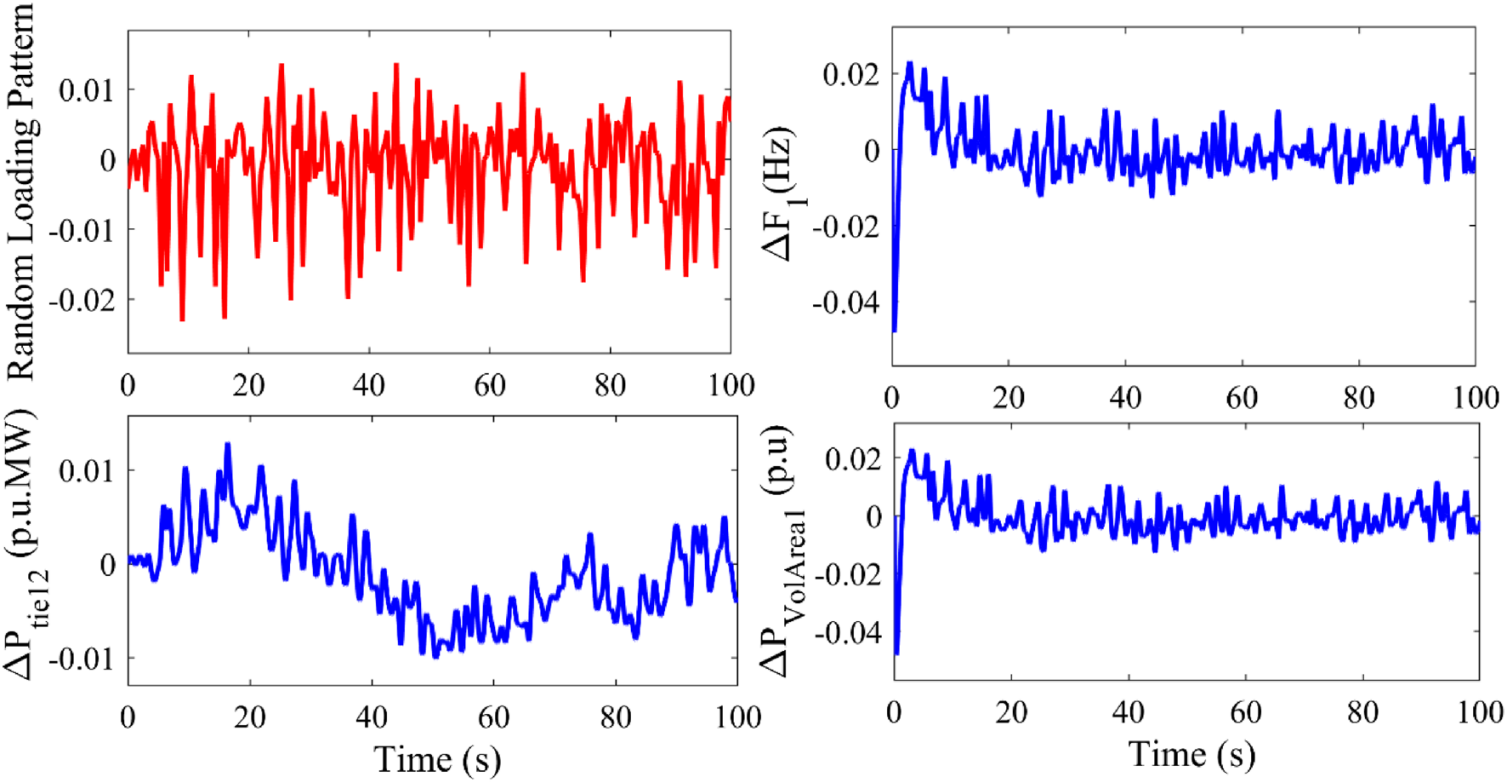
Dynamic system responses with the implementation of an electric spring for random disturbances in Area 1.

In the previous case study, implementation of electric spring has stabilized the terminal voltage in AVR loop in both control areas followed by frequency deviation in Area 1 and Area 2 when the disturbances considered in Area 1 is step load perturbation. In the present case study, the proposed system with implementation of electric spring is now investigated for random loading perturbation The proposed cascade controller 2DOF PID – PID controller is utilized optimized by Golden Jackal Optimization for optimizing the controller parameters whose tuned parameters are tabulated in column 6 in Table 4. With the tuned parameters, system dynamic responses are plotted and are depicted in FIGURE 9. Only the relevant responses such as the random loading pattern, frequency deviation in Area 1, tie-power deviation in Area 1 and Area 2 and the voltage deviation in Area 1 (generator terminal) are presented.

The figures infer that even with random loading perturbation, which is equivalent to random load disturbances at every point in time, the proposed controller has successfully mitigated the area control error by mitigating the frequency, tie-power, and voltage deviation within safe limits.

### Case study 6: investigation with the performance index for different types of algorithms

**Fig. 10:**
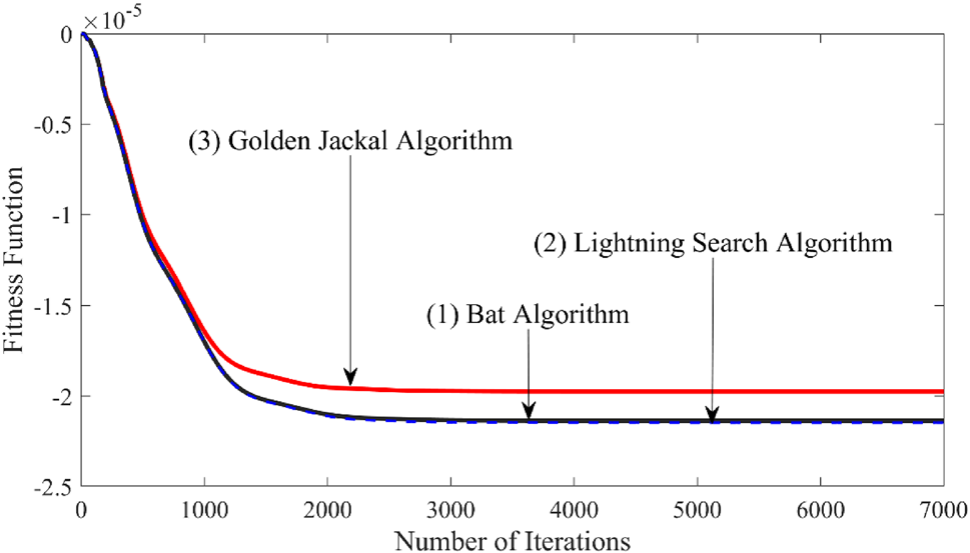
Comparison of the performance index of three algorithms with a nominal system.

The present study investigates the performance index or fitness function of three algorithms: the Bat Algorithm^27^the Lightning Search Algorithm^4^and the Golden Jackal Algorithm^28^ with the nominal model. The nominal model includes the ALFC loop only with the proposed controller and AC interconnection. It is considered that the performance index among the three algorithms can be established easily with the nominal model. For fair comparison, the population size is fixed at 50. The other parameters are considered as those in Ref. 4, Ref. 27, and Ref. 28 for the three algorithms, respectively. The nominal model secondary controller gains and parameters are tuned with the algorithms one by one, and the performance index vs. several iterations behaviour is plotted and compared. The compared figure is depicted in FIGURE 10. The fitness function or the performance index (ITAE) among all three algorithms states that the value is lower for the Golden Jackal algorithm, and it converges almost at a similar time as the others. Hence, the proposed Golden Jackal Optimization is a robust algorithm for tuning the controller gains and parameters. The controller gains and parameters are not tabulated for the three algorithms (not in the scope of the investigation).

## Conclusion

A coordinated frequency and voltage control scheme is implemented in a two-area thermal gas system with a dish Stirling solar thermal plant and a wind turbine generator in each area. One control area is half of the capacity of another control area. A novel cascade controller, namely 2DOF PID-PID, is utilized in both ALFC and AVR coordinated control areas. A powerful Golden Jackal Optimization algorithm is employed for tuning the controller parameters in the two control areas. An illustration of the concept of an electric spring is demonstrated for stabilizing the voltage deviation across the generator terminals of the AVR loop in both control areas. Investigations on terminal voltage deviations are carried out with step load and random loading patterns. Out of comparisons among various performance indices, ITAE is observed to be the best PI for the proposed system with the proposed cascade controller. Also, for further demonstration of the effectiveness of the proposed algorithm, a comparison of different algorithms proves the proposed one to be superior in terms of performance index and iterations. While investigating the best controller for the secondary loop as well as controller for the AVR loop, comparison of controller among PID, 2 DOF PID and the proposed 2 DOF PID – PID suggests that implementing the cascade 2 DOF PID – PID controller in presence of a renewable energy integrated system can achieve the target of zero area control error. Results show that the coordinated control of frequency and voltage in the presence of a cascade 2 DOF PID–PID controller is successful in mitigating the terminal voltage deviation across the generator along with frequency deviations in both control areas. The scope of the electric spring connected in association with an AC/DC parallel link in the two-area power system is also examined for the system with the proposed 2 DOF PID–PID controller. Future scope includes incorporating an electric spring to enhance the power quality as a harmonic suppressor, and nonlinear modeling of the electric spring to contribute to a system comprising inverter-based resources.

## Data Availability

The data that support the findings of this study are available from the corresponding author upon reasonable request.

## Appendix 1: System parameters depicting thermal, gas, wind turbine generator and dish stirling solar thermal plant.

**Table:**

Sl. No.	Plants	Gains and time constants	
1.	Thermal plant	Governor Gain, K_G_ = 1	
		Governor Time Constant, T_G_ = 0.08 s	
		Turbine Gain, K_T_ = 1	
		Turbine Time Constant, T_T_ = 0.3 s	
		Reheat Gain, K_RG_ = 5	
		Reheat Time Constant, T_RG_ = 10 s	
2.	Gas plant	Speed governor lead time constant, X_G_ = 0.2	
		Speed governor lag time constant, Y_G_ = 1.1 s	
		Valve Positioner Constant, a_G_ = 0.1	
		Valve Positioner Constant, c_G_ = 1	
		Valve Positioner Constant, b_G_ = 0.049	
		Combustion reaction time delay, T_CR_ = 0.01 s	
		Fuel time constant, T_F_ = 0.239 s	
		Compressor discharge volume time constant, T_CD_ = 0.2 s	
		Governor Gain, K_G_ = 0.130438	
3.	Wind turbine generator	K_WTG_ = 1, T_WTG_ = 1.5 s	
4.	Dish stirling solar thermal plant	K_DSTS_ = 1, T_DSTS_ = 5 s	
5.	AVR system	K_AMP_ = 1.11, T_AMP_ = 2 s, K_EXC_ = 0.4, T_EXC_ = 1 s, K_SEN_ = 1, T_SEN_ = 0.05 s, K_f_ = 0.75, T_f_ = 1.4 s, K_1_ = 01, K_2_ = 0.02, K_3_ = 0.2, K_4_ = 0.4, K_5_ = 1	
